# Interactive effects of chemical and biological controls on food-web composition in saline prairie lakes

**DOI:** 10.1186/2046-9063-8-29

**Published:** 2012-11-27

**Authors:** Ryan N Cooper, Björn Wissel

**Affiliations:** 1 Environmental Quality Analysis Laboratory, Department of Biology, University of Regina, 3737 Wascana Parkway, Regina, Saskatchewan S4S 0A2, Canada

**Keywords:** Lake food-web, Diversity, Fish, Invertebrates, Salinity, Winter kill

## Abstract

Salinity is restricting habitatability for many biota in prairie lakes due to limited physiological abilities to cope with increasing osmotic stress. Yet, it remains unclear how salinity effects vary among major taxonomic groups and what role other environmental parameters play in shaping food-web composition. To answer these questions, we sampled fish, zooplankton and littoral macroinvertebrates in 20 prairie lakes (Saskatchewan, Canada) characterized by large gradients in water chemistry and lake morphometry. We showed that salinity thresholds differed among major taxonomic groups, as most fishes were absent above salinities of 2 g L^-1^, while littoral macroinvertebrates were ubiquitous. Zooplankton occurred over the whole salinity range, but changed taxonomic composition as salinity increased. Subsequently, the *complexity of fish community* (diversity) was associated with large changes in invertebrate communities. The directional changes in invertebrate communities to smaller taxa indicated that complex fish assemblages resulted in higher predation pressure. Most likely, as the complexity of fish community decreased, controls of invertebrate assemblages shifted from predation to competition and ultimately to productivity in hypersaline lakes. Surprisingly, invertebrate predators did not thrive in the absence of fishes in these systems. Furthermore, the here identified salinity threshold for fishes was too low to be a result of osmotic stress. Hence, winterkill was likely an important factor eliminating fishes in low salinity lakes that had high productivity and shallow water depth. Ultimately, while salinity was crucial, intricate combinations of chemical and biological mechanisms also played a major role in controlling the assemblages of major taxonomic groups in prairie lakes.

## Introduction

The formation of saline lakes is favored in endorheic (interior) drainage basins that are located in semi-arid and sub-humid climates where evaporation exceeds precipitation [1]. Saline lakes are classified according to salinity (in g L^-1^ of total dissolved solids, TDS; [2] as fresh (< 0.5 g L^-1^), subsaline (0.5 - 3 g L^-1^), hyposaline (3–20 g L^-1^), mesosaline (20–50 g L^-1^), and hypersaline (> 50 g L^-1^). Further distinctions are made due to ion composition and the degree of permanence of these lakes [3,4]. Despite the limited awareness of saline lakes, these systems are found on all continents and account for almost half of the total volume of inland surface standing water on Earth [1,5]. Across the Canadian prairies, saline lakes are of particular interest as all five salinity classes are represented within a small geographic area [6,7]. Lakes are either shallow (< 3 m) and polymictic or deeper (> 5 m) and meromictic [1,4]. Furthermore, lake size and depth ranges over several orders of magnitude, and gradients of water chemistry parameters, such as nutrients, dissolved carbon and chlorophyll *a* are large as well [1,7].

Previous studies of species assemblages in saline systems illustrated that food-web composition can be considerably different among lakes. For instance, mesosaline lakes, e.g., altiplano lakes of the Andes [8] and prairie lakes in Saskatchewan [1,9], can have extensive food webs with diverse pelagic and littoral invertebrate communities and fish assemblages that include multiple trophic levels. Conversely, very simplified food webs are found in many hypersaline lakes (e.g. Lake Nakuru, Kenya; Great Salt Lake, Utah) where algal communities are often restricted to a small number of species, and higher trophic levels are limited to few large-bodied crustaceans, such as *Artemia sp.*, and birds [10]. The decline in species richness and changes in taxonomic composition have been well described in association with increasing salinity [1,3,11,12]. Zooplankton diversity declines at higher salinities, yet, taxa occur over a very large salinity gradient with species assemblages changing from freshwater to salinity-tolerant and finally halophilic species [9,13]. In contrast, abundance and composition of littoral and benthic macroinvertebrate communities remain largely unchanged until about 20 g L^-1^, beyond which diversity declines quickly ([1,14]; [15,16]). For fishes, richness and abundance are impoverished above salinities of 5–8 g L^-1^, with most meso- and hypersaline lakes being fishless [1,3,4,11].

Many previous studies on species distributions in saline lakes focused on salinity, but it is largely unknown how other environmental parameters (ionic composition, nutrients, trophic interactions, morphometry) impact species composition, what the underlying mechanisms are, and if the impacts of these parameters are universal or taxon-specific. Furthermore, identifying the mechanisms that control food-web structure in saline lakes will be important to anticipate the potential impacts of climate change on these systems. Global climate change scenarios predict that the Canadian plains will experience more droughts in the future ([17,18]; [19], leading to severe water shortages and higher salinities. Increased water-use by agriculture, municipalities and industries will likely add additional strains to many already stressed aquatic environments, and endorheic lakes may be disproportionally impacted due to their high sensitivity to hydrologic changes [18,20]. The potential impacts of future climate change on prairie lake ecosystems may be evaluated using a space-for-time approach, with the existing large spatial diversity of environmental conditions across prairie lakes representing future temporal changes within individual lakes [21].

Here we present the results of a 20-lake, two-year survey that evaluated environmental parameters and taxonomic assemblages of zooplankton, littoral macroinvertebrates and fishes to quantify the importance of chemical and biological parameters for controlling these communities. This study is founded on a prior analysis of zooplankton composition in 70 prairie lakes [13], which identified a hierarchical control where salinity was the principal correlate of changes in taxonomic composition, while nutrients and water depth provided secondary mechanisms structuring zooplankton composition. Furthermore, a concurrent analysis of food-web complexity in these 20 lakes using stable-isotope based metrics, showed strong declines in complexity not only with salinity but also with the loss of piscivorous fishes. The goals of this study were to 1) evaluate if taxonomic assemblages of zooplankton, littoral invertebrates and fishes differ in their sensitivities and thresholds to environmental controls, 2) identify the potential importance of biotic interactions, and 3) predict the potential impacts of future climate change on these systems.

## Methods and materials

### Study area

**Figure 1:**
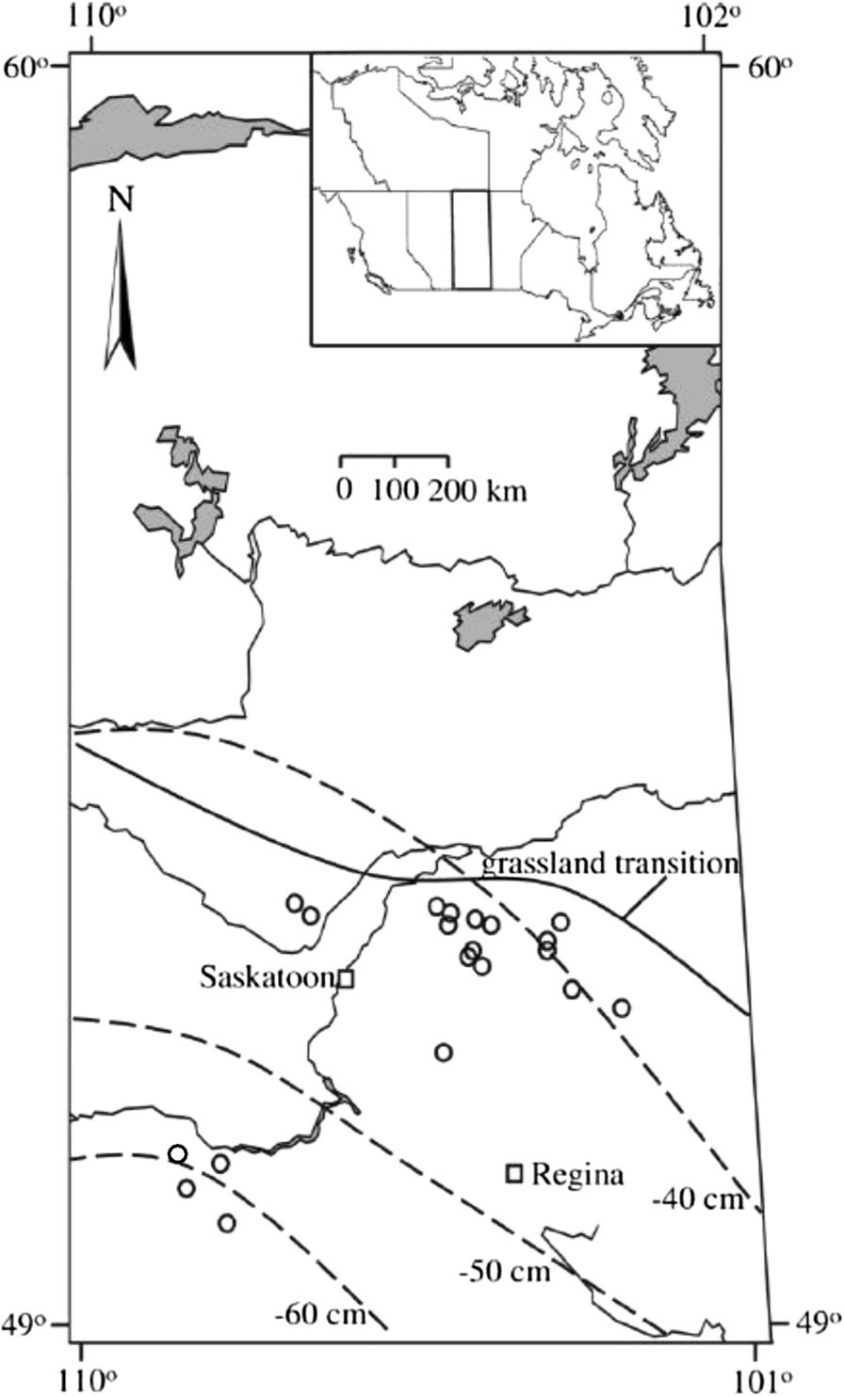
**Location of the sampling sites in the northern Great Plains of Saskatchewan, with sample sites as open circles and major cities as open squares. **Dashed lines indicate long-term isopleths of total precipitation minus total potential evapotranspiration (cm yr^-1^) [22]. Solid line denotes grassland transition. All lakes are in endorheic drainage basins.

The study was conducted in 20 lakes across southern Saskatchewan, Canada. This prairie region (49-53°N, 103-109°W) is defined by a transition from a semi-arid climate in the southwest to a sub-humid climate in the northeast (Figure 1). Mean summer (May to September) temperatures are 13-15°C and mean spring and summer precipitation during this period varies from ~118 mm in the southwest to ~240 mm in the northeast [7,22]. Evapotranspiration in this area is high and may exceed precipitation by 40–60 cm yr^-1^[6]. All study lakes were in endorheic drainage basins with snow melt and surface run-off as main inflows [20]. Hydrological connections among individual lakes exist but flow is limited to high run-off periods and unusually wet years. The 20 study lakes have been sampled for water chemistry parameters and zooplankton since 2002 [7,20], and represent similar large gradients in environmental conditions as a broader 70-lake survey that was conducted in August 2004 [7,13]. Yet in comparison to the previous study, lakes were deeper (> 3 m maximum depth), providing a well defined pelagic area that is important for a diverse fish community [23,24], and calcium concentrations remained above 10 mg L^-1^, avoiding potentially confounding affects of low species diversity due to calcium limitation [25,26], [13].

### Sampling and analyses

Each lake was sampled in June and August of 2007 and 2008, with the exception of Rabbit (not sampled in June 2008) and Middle Lakes (not sampled in August 2008), which were occasionally inaccessible due to poor road conditions. Dissolved oxygen (mg L^-1^), salinity and total dissolved solids (g L^-1^), specific conductivity (μS cm^-1^), water temperature (°C), and pH were measured throughout the water column in 0.5 m intervals in shallow lakes (< 5 m), or in 1 m intervals in deeper lakes (> 5 m) using a YSI multi probe (model 556). Water transparency was measured with a 20 cm black and white Secchi disk. We used a tube sampler to collect integrated, prescreened (80-μm mesh) water samples for water chemistry analyses (total Kjeldahl nitrogen (TKN), nitrate (NO_3_), ammonium (NH_4_), total phosphorus (TP), soluble reactive phosphorus (SRP), dissolved inorganic carbon (DIC), dissolved organic carbon (DOC), calcium (Ca) and chlorophyll *a* concentration (Chl *a*). The tube sampler was suspended into the water column either down to 6 m for deeper polymictic lakes, down to 0.5 m above bottom sediments for shallower polymictic lakes, or down to 0.5 m above the monimolimnion for meromictic lakes to prevent the inclusion of potentially anoxic, nutrient-rich water layers that were not accessible to the studied biota. For Chl *a*, integrated samples were filtered onto prewashed GF/C filters and stored at −10°C until extraction with an acetone-methanol–water (80:15:5 by volume) solution using standard trichromatic methods [27]. For water chemistry, samples were filtered through a 0.45 μm filter and stored at 4 °C until analyses. Quantification of NO_3_, NH_4_, TKN, SRP, TP (all μg L^-1^) and Ca (mg L^-1^) were performed at the University of Alberta Water Chemistry Laboratory following standard procedures [7,28,29]. DIC and DOC (both mg L^-1^) were analyzed on a Shimadzu TOC Analyzer 5000A at the Environmental Quality Analysis Laboratory (EQAL) at the University of Regina.

In all lakes that potentially supported fish (TDS < 20 g L^-1^), the near-shore fish community was sampled using a beach seine (2 m x 30 m, 10-mm mesh). Twice at each sampling date, the beach seine was pulled out perpendicular to shore, slowly brought back creating a semi-circle, and carefully retrieved to prevent escapement of fish. Captured fish were euthanized with buffered tricaine methonesulphonate (MS-222; [30,31]) and kept on ice until return to the laboratory, where they were frozen. Information on presence and absence of pelagic fishes was supplemented with data from the Saskatchewan Ministry of Environment (2002-2007; [32]), which provided information on bi-annual multi-panel gill-net surveys of pelagic fish communities. Reported species that occurred in freshwater and subsaline study lakes (Shannon, Lenore, Fishing, Humboldt, Kipabiskau, Pelletier, Wakaw, Edouard) included northern pike (*Esox lucius*), walleye (*Sander vitreus*), yellow perch (*Perca flavescens*), whitefish (*Coregonus clupeaformis*) and common sucker (*Catostomus commersonii*). Furthermore, six of the study lakes (Shannon, Lenore, Fishing, Humboldt, Kipabiskau, Pelletier) have been regularly stocked with walleye [33]) as this species is lacking sufficient spawning habitat in these lakes. Ultimately, we are confident that, combined with the supplementary information, our survey generated reliable information on presence/absence of the ambient fish species in the study lakes. Subsequently, fish species composition was characterized by complexity (*high complexity* - communities included piscivores, planktivores and benthivores; *low complexity* - assemblages lacked piscivores, and *fishless*), with complexity largely representing the number of trophic levels within the fish community.

Pelagic invertebrates were collected using 80-μm (30 cm diameter) and 500-μm (50 cm diameter) plankton nets, respectively, which were towed vertically from maximum depth to the surface at the deepest point of the lake. Samples from each net were preserved with an ethanol-sucrose solution for species abundances (ind. L^-1^) and taxonomic analyses. Individuals were identified to species for anostraca, cladocerans, and copepods, to genus for rotifers, and to order for aquatic insects and other crustaceans [34-36]. For the enumeration of larger taxa (anostraca, aquatic insects, amphipods) the whole sample was analyzed, while for cladocerans, copepods and rotifers sub-samples (10-20%) were analyzed until at least 100 specimens per taxa were enumerated.

Littoral macroinvertebrates were collected from near-shore areas in depths < 1m using a large sweep net (500-μm mesh size). Samples were pooled from different littoral habitats (gravel, sand, mud, and macrophyte stands, if available) that were each sampled for approximately 10 minutes. Samples were preserved in ethanol to estimate presence/absence, and specimens were identified to order [35] using a dissecting microscope. Densities (ind. m^2^) were not calculated, as this would have required a much larger sampling effort including the quantification of individual habitat types combined with stratified sampling for each lake. Littoral macroinvertebrates in pelagic habitats were evaluated based on their presence/absence in the 500-μm (50 cm diameter) plankton net hauls (see above).

### Statistical analysis

Because we relied on presence/absence data for fishes and littoral macroinvertebrates, we felt that these communities were more accurately represented by 2-year averages rather than by individual sampling events. Furthermore, previous work on zooplankton communities in the 20 study lakes showed that seasonal and inter-annual variability within lakes was small (< 3% of total explained variability) relative to the among lake differences [13]. Accordingly, we conducted all subsequent statistical analyses on averages over the two-year sampling period. For all analyses, species abundances and environmental variables, except pH, were log_10_ (x + 1) transformed to obtain normality, and taxa occurring in only one lake were omitted.

To analyze the associations among environmental parameters and quantify the amount of variability explained by linear gradients we performed a principal component analysis [37] (PCA, CANOCO version 4.5). Subsequently, we performed stepwise multiple linear regressions on taxonomic richness (# of taxonomic groups) for fishes, zooplankton, and littoral macroinvertebrates to identify how taxonomic richness changed along environmental gradients. For zooplankton, we further explored this relationship by analyzing species diversity as Shannon Index (H) and Shannon Evenness Index (E_H_) [38].

To identify those environmental parameters that significantly contributed to variation in taxonomic compositions of prairie lakes we conducted Canonical Correspondence Analyses (CCA, CANOCO version 4.5). CCAs were performed on presence/absence data for littoral macroinvertebrates and fishes, and on mean abundances for zooplankton. CCA is a direct gradient analysis technique [39] that performs well with nonlinear and unimodal species-environmental relationships [40] and is relatively insensitive to data transformation protocols [41,42]. All environmental variables (salinity, TDS, conductivity, NO_3_, TKN, NH_4_, SRP, TP, DIC, DOC, Ca, depth, surface area, elevation, Secchi depth, Chl *a*, and pH) were included into the initial CCA, but to avoid multicollinearity three highly correlated variables were omitted from the analyses (TDS, conductivity, and SRP). For CCAs of zooplankton and littoral macroinvertebrates communities we added complexity of fish assemblage (*high, low, fishless*) as a biological predictor. Using forward selection with 1000 Monte Carlo iterations, we identified environmental variables that significantly affected the taxonomic compositions within the study lakes. Significance of CCA axes was determined using the eigenvalues of the identified environmental parameters as test statistics.

## Results

### Environmental parameters

**Figure 2:**
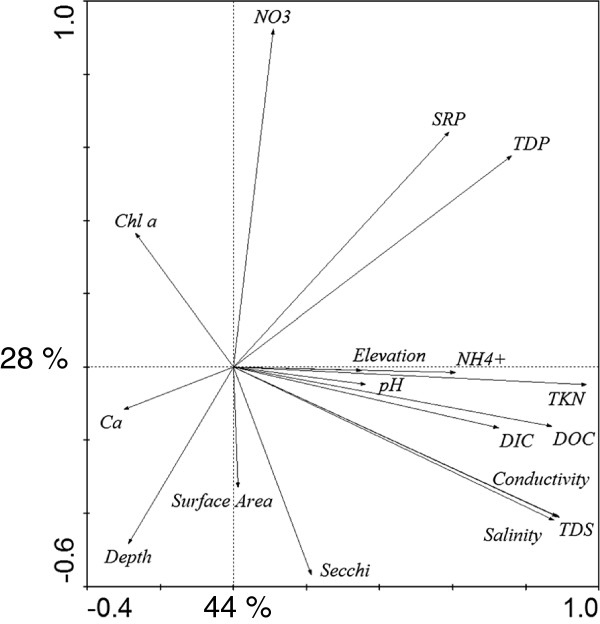
**Relationships among environmental parameters for the 20 study lakes using a principle components analysis (PCA). **PCA axes 1 and 2 explained 44 and 28% of the variability, respectively. PCA explained 83% of the cumulative variance on all four axes.

Mean values for water chemistry and lake morphometry varied several fold among the 20 study lakes (Table 1). The lakes showed a large variability in nutrient levels, such as NH_4_ (median 71, range: 31 to 7,585 μg L^-1^), TP (median 89, range: 9 to 636 μg L^-1^), and TKN (median 1671, range: 746 to 14,356 μg L^-1^). Salinity ranged from freshwater to hypersaline lakes, with TDS between 0.4 to 64 g L^-1^. Water depth included shallow-mixed (3 m) systems and deep-meromictic lakes (30 m). Lake size varied over two orders of magnitude, from 0.7 to 60.7 km^2^ (median 4.7 km^2^). pH varied relatively little among lakes, ranging from 8.1 to 8.9 (median 8.6). PCA explained 83% of the variance in environmental parameters among the 20 study lakes (Figure 2), with PCA axes 1, 2 and 3 accounting for 44, 28, and 11% of the variability, respectively. Salinity, conductivity, TDS, DOC, DIC, TKN, NH_4_, pH, and elevation were positively associated with PCA axis 1, while Chl *a*, Ca and water depth had negative associations. NO_3_-SRP-TP and Secchi depth-surface area-water depth were positively and negatively associated with PCA axis 2, respectively. Water depth was negatively correlated with PCA axis 3. Variability in Chl *a* among lakes was only weakly associated with water chemistry parameters. Yet, after removal of Lake Kipabiskau (which received significant nutrient inputs in 2006) from stepwise linear regression analysis and calculating depth-weighted Chl *a* concetrations, we identified significant positive and negative associations of Chl *a* with TKN and salinity (p < 0.01, r^2^ = 0.32), respectively.

**Table 1:** Chemical and physical characteristics of the 20 study lakes

Lake	Latitude °N (dd)	Longitude °W (dd)	Elevation (masl)	Surface (km^2)^	Depth (m)	Cond. (μS cm^-1^)	TDS (g L^-1^)	Secchi (m)	Chl a (μg L^-1^)	pH	TKN (μg L^-1^)	NO_3_ (μg L^-1^)	NH_4_^+^ (μg L^-1^)	TP (μg L^-1^)	SRP (μg L^-1^)	Ca (mg L^-1^)	DIC (mg L^-1^)	DOC (mg L^-1^)	Mixis Type
**Eduoard**	52.38	104.33	580	1.0	5.1	0.6	0.4	1.7	25	8.3	1488	278	229	136	91	49	42	21	P
**Pelletier**	49.98	107.93	825	2.0	7.4	0.8	0.5	1.7	17	8.6	746	4	44	12	8	23	74	15	P
**Kipabiskau**	52.56	104.20	522	5.2	7.3	0.8	0.6	1.8	7	8.3	1159	287	31	173	130	83	50	26	P
**Lenore**	52.50	104.98	537	10.0	8.8	1.5	1.1	2.0	15	8.5	809	10	45	9	2	59	59	26	P
**Humboldt**	52.15	105.10	544	19.1	5.3	1.6	1.1	1.1	25	8.5	1469	243	85	310	206	94	49	23	P
**Clair**	51.98	104.05	524	1.2	3.1	2	1	0.9	22	8.7	1317	221	32	33	10	73	49	25	P
**Wakaw**	52.66	105.58	511	10.7	7.1	3	2	1.9	12	8.1	1066	17	134	9	5	164	39	21	P
**Shannon**	52.63	105.43	549	1.0	7.6	3	2	1.7	11	8.8	1420	224	59	40	15	65	65	24	P
**Fishing**	51.83	103.50	529	32.1	11.4	3	2	2.6	14	8.4	1104	2	37	9	7	99	57	23	P
**Rabbit**	52.60	107.00	504	4.6	4.0	8	6	1.2	10	8.7	1734	154	30	210	153	76	99	36	P
**Charron**	52.40	104.33	556	4.0	7.3	9	6	2.0	25	8.8	2158	153	75	145	79	67	90	33	P
**Arthur**	52.56	105.43	541	2.9	4.3	13	9	3.3	6	8.5	2448	40	67	84	16	186	68	50	P
**Middle**	52.56	105.16	534	5.9	5.0	15	11	3.9	21	8.7	3191	240	77	95	6	103	95	55	M
**Deadmoose**	52.31	105.16	539	10.9	29.3	15	11	2.5	11	8.7	1788	13	113	43	9	95	65	30	M
**Waldsea**	52.28	105.20	533	4.7	11.0	16	12	2.7	10	8.6	1608	6	67	23	12	226	55	42	M
**Redberry**	52.71	107.15	502	60.7	11.3	18	13	3.2	4	8.7	2011	10	32	27	19	61	131	43	P
**Antelope**	50.28	108.40	701	13.8	3.3	19	15	1.1	14	8.9	4816	117	281	126	63	37	189	72	P
**Success**	50.48	108.01	715	0.7	14.0	28	20	3.6	9	8.9	12657	22	7585	134	115	14	350	49	M
**L. Manitou**	51.75	105.50	493	12.8	4.9	58	44	1.9	16	8.5	5741	11	133	321	47	50	91	112	P
**Snakehole**	50.30	108.28	858	1.3	2.9	71	64	1.8	7	8.5	14356	43	187	636	126	53	109	318	P

Values, except elevation and surface area, are means of the four sampling periods (June 2007, August 2007, June 2008, and August 2008; Rabbit Lake was not sampled in June 2008 and Middle Lake was not sampled August 2008). Lakes are sorted by increasing salinity. P and M represent polymixis and meromixis, respectively.

*Fish* – Fish were present in 12 of the 20 study lakes and a total of 10 species were collected: yellow perch, walleye, northern pike, fathead minnow (*Pimephales promelas*), pearl dace (*Margariscus margarita*), spottail shiner (*Notropis hudsonius*), nine-spine stickleback (*Pungitius pungitius*), brook stickleback (*Culea inconstans*), lake whitefish, and common sucker. According to the grouping by complexity of fish assemblage, seven, five and eight lakes were associated with *high complexity* (Pelletier, Kipabiskau, Lenore, Humboldt, Wakaw, Shannon, Fishing Lakes), *low complexity* (Edouard, Clair, Redberry, Deadmoose, Waldsea Lakes) and *fishless* (Rabbit, Charron, Arthur, Middle, Antelope, Success, Little Manitou, Snakehole Lakes), respectively.

Excluding fishless lakes, taxonomic richness of fishes ranged from 1 to 7 species per lake and multiple linear regressions determined that salinity and NO_3_ had significant negative associations with fish species richness (r^2^ = 0.75; Table 2). With the exception of nine-spine stickleback in Waldsea (11.9 g L^-1^ salinity), Redberry (12.8 g L^-1^ salinity), and Deadmoose (10.7 g L^-1^ salinity) Lakes, fishes were not captured above 2 g L^-1^ salinity, but occurred in all lakes below this point. Nevertheless, extensive fish communities were absent from two shallow freshwater lakes (Clair and Eduoard), which were inhabited only by fathead minnows or sticklebacks.

**Table 2:** **Stepwise multiple linear regression results of environmental, physical, and biological factors influencing Shannon Index (H), Shannon Evenness Index (E**_**H**_**), and taxonomic richness for zooplankton, littoral macroinvertebrates and fishes**

**Biodiversity metric**	**Regression**	**r**^**2**^_**adj.**_
H	−0.8 salinity + 0.6 Chl *a* - 0.4 SRP - 0.1 NH_4_^+^	0.85
E_H_	−0.7 salinity - 0.5 area	0.74
Zooplankton richness	−0.7 salinity + 0.4 Chl *a* - 0.3 NO_3_^-^ + 0.2 elevation	0.82
Littoral macroinvertebrate richness	−0.7 salinity	0.48
Fish richness	−0.8 salinity - 0.2 NO_3_^-^	0.74

H and E_H_ are calculated only for zooplankton.

**Figure 3:**
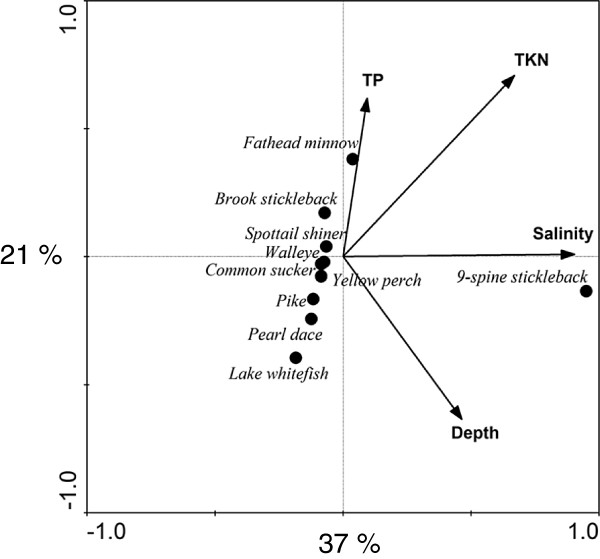
**Canonical Correspondence Analysis (CCA) depicting the relationship between fish species distribution and environmental variables. **All variables (lake morphometry, environment, water chemistry) were analyzed, but only those that were statistically significant (p < 0.05) were retained. The x- and y-axes explain 37% and 21% of the variation, respectively. Conductivity and salinity were omitted due to the strong correlation to TDS.

CCA showed that fish species composition was significantly associated with salinity, TKN, TP and depth (Figure 3). CCA axes 1, 2 and 3 explained 37, 21 and 4% of the variance, respectively. Salinity had a positive correlation to CCA axis 1 and no relationship to CCA axis 2, TKN had positive correlations to CCA axes 1 and 2, and TP and depth were associated positively and negatively with axis 2, respectively (Figure 3). CCA determined that fish species composition were predominantly effected by salinity and secondary by depth and measures of productivity (Figure 3). Of the fish species observed, nine-spine stickleback were most halotolerant, while pike, lake whitefish, spottail shiner, yellow perch, walleye, and pearl dace were found at low salinities and low nutrients. Three-spine sticklebacks, common suckers, and fathead minnows were associated with low salinities and intermediate to high nutrient concentrations.

### Zooplankton

Taxonomic analysis of the 20 study lakes revealed 20 taxa of zooplankton, including six genera of rotifers (*Asplanchna*, *Keratella*, *Polyarthra*, *Collotheca*, *Kellicottia*, and *Trichocera*), nine species of cladocerans (*Leptodora kindtii*, *Daphnia pulex*, *D. galeata mendotae*, *D. rosea*, *D. similis, D. retrocurva, Diaphanosoma birgei*, *Ceriodaphnia lacustris*, *Bosmina longirostris*), one branchiopod (*Artemia franciscana*), and four taxa of copepods (*Leptodiaptomus sicilis*, *Diacyclops thomasi, Hesperodiaptomus nevadensis*, and harpacticoids). The single occurrences of *D. retrocurva* and *L. kindtii* resulted in their exclusion from statistical analysis. Additionally, we encountered the following six littoral / benthic taxa in our pelagic samples: *Gammarus pulex*, *Hyalella azteca*, hydracarinids, corixids, zygoterans and chironomids.

Within the study lakes, taxonomic richness of pelagic zooplankton ranged from 3 to 18 species, with lower taxonomic diversity in hypersaline lakes. Zooplankton richness was negatively associated with salinity with an r^2^ of 0.71 (Table 2). For the Shannon Diversity Index (H) salinity was the most influential factor with NH_4_, SRP, and chlorophyll *a* concentrations contributing equally to explaining subsequent variability (r^2^ = 0.85; Table 2). E_H_ was negatively influenced by salinity and surface area (r^2^ = 0.74).

CCA revealed that the zooplankton community was significantly associated with salinity, fish assemblage, elevation, and nutrient content (NO_3_ and NH_4_) (Figure 3). CCA axes 1, 2 and 3 respectively explained 26, 15 and 10% of the taxonomic variation. Salinity showed a strong correlation to CCA axis 1, while NH_4_ and elevation had weaker positive correlations. In contrast, fish assemblage and NO_3_ had negative relationships to CCA axis 1. CCA axis 2 was most strongly influenced by fish assemblage (*high complexity*: negative association, *fishless*: positive association), with weaker positive correlations to NO_3_, NH_4_, and salinity. CCA axis 3 had a strong positive correlation to NH_4_, with a weaker positive association with elevation, and a negative correlation to NO_3_^.^ Species groupings were determined by salinity along CCA axis 1, fish assemblage along CCA axis 2, and nutrients on CCA axis 3. *A. franciscana* and harpacticoids were associated with highest salinities, independent of other environmental parameters, and occurred in *fishless* lakes. Larger zooplankton taxa and several littoral organisms (*L. sicilis*, chironomids, Zygoptera, corixids, *Asplanchna*, *H. nevadensis, H. azteca*, *D. similis,* and *D. pulex*) were found at intermediate salinities and in freshwater lakes that were *fishless* or had *low complexity*. Small zooplankton taxa (*D. rosea*, *D. galeata mendotae, D. birgei*, *Diacyclops thomasi,* hydracarina, *Keratella*, *B. longirostris*, *Kellicottia, Collotheca*, and *C. lacustris*) were characteristic of low salinities and co-occurred with fishes (positive association with *high complexity*).

### Littoral macroinvertebrates

Eighteen taxa of macroinvertebrates were encountered in the littoral zones of the 20 study lakes, including collectors/scrapers: Turbellaria sp., *H. azteca*, *G. lacustris*, corixids, Culicidae, chironomids, Ephemeroptera, ephydrids, and gastropods; predators: Hydrophilidae, Hirudinaea, Hydracarina, notonectids, Dytiscidae, Zygoptera and crayfish; and shredders: Halipidae and Trichoptera. Taxonomic richness ranged from 1 to 13, with only one species occurring in hypersaline lakes. Multiple linear regressions determined that salinity was most influential for richness, but compared to zooplankton this association was fairly weak (r^2^ = 0.48; Table 2).

**Figure 4:**
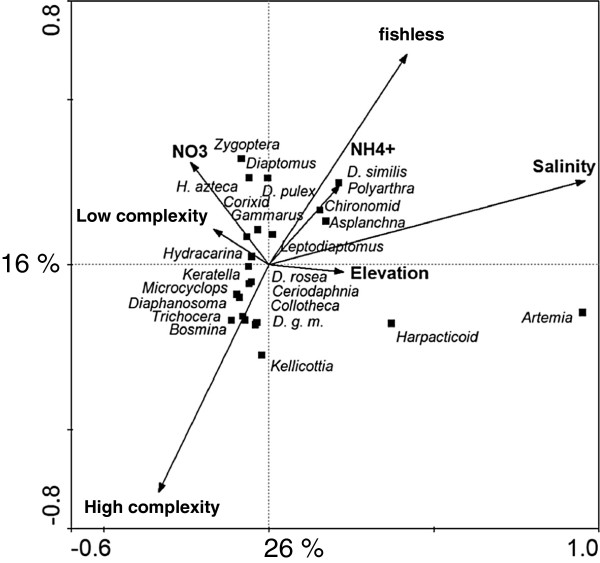
**Canonical Correspondence Analysis (CCA) of the relationship between zooplankton taxa and environmental variables. **All variables (lake morphometry, environment, water chemistry, and land use) were analyzed, but only those that were statistically significant (p < 0.05) were retained. High complexity (piscivorous and planktivorous/benthivorous fishes), low complexity (planktivorous / benthivorous fishes) and fishless (no fishes) indicates the complexity of fish assemblages. The x- and y-axes explain 26% and 16% of the variation, respectively. Conductivity and salinity were omitted due to the strong correlation to TDS.

CCA illustrated that the taxonomic composition was significantly associated with salinity, fish assemblage, DOC, and Chl *a* (Figure 4). CCA axes 1, 2 and 3 respectively explained 34, 13 and 7% of the variability. Salinity and DOC had a strong positive correlation to CCA axis 1, while high complexity of fish assemblage and Chl *a* had negative correlations. CCA axis 2 had strong and weak negative correlations to *high complexity* of fish assemblage and Chl *a*, respectively, and weak positive correlations to salinity and DOC. CCA axis 3 had a strong positive correlation to Chl *a* and weak correlations to DOC and salinity. Species groupings were determined by salinity gradients on CCA axis 1 and fish assemblage on CCA axis 2. Ephydrids were associated with the highest salinities, and were unaffected by fish assemblage and Chl *a*. Hydrophilids, chironomids, corixids, Zygoptera, and *H. azteca* were found at intermediate values for salinity and were only slightly affected by fish assemblage, whereas Culicidae, gastropods, hydracarinids, dytiscids, Hirudinea, Turbellaria, crayfish, Trichoptera, Ephemeroptera, caenids, and *G. lacustris* were characteristic of lakes with low salinity and high complexity of fish assemblage.

**Figure 5:**
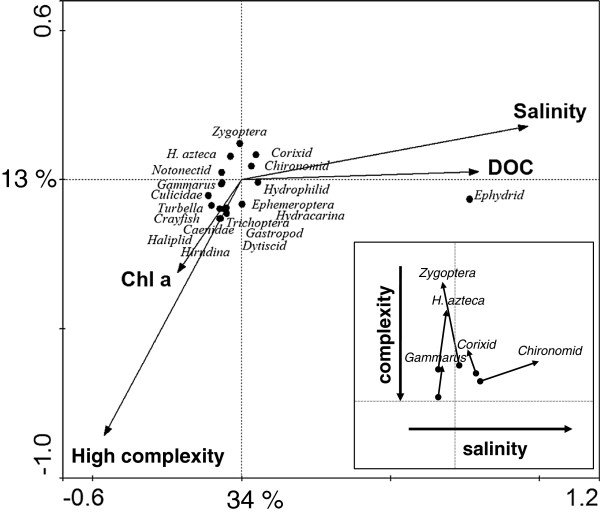
**Canonical Correspondence Analysis (CCA) showing the relationship between littoral macroinvertebrate taxa and environmental variables. **All variables (lake morphometry, environment, water chemistry, and land use) were analyzed, but only those that were statistically significant (p < 0.05) were retained. High complexity (piscivorous and planktivorous / benthivorous fishes), low complexity (planktivorous/benthivorous fishes) and fishless (no fishes) indicates the complexity of fish assemblages. The x- and y-axis explain 34% and13% of the variation, respectively. Conductivity and salinity were omitted due to the strong correlation to TDS. Insert. Canonical Correspondence Analysis (CCA) using the same variables and data as in the large figure, identifying those macroinvertebrates that were captured in both littoral and pelagic samples. Locations of taxa captured in littoral samples are represented by circles and pelagic locations are represented by triangles. The changes in location between littoral and pelagic captures represent the changes in environmental conditions. Amphipods, corixids, and Zygoptera all moved up along the y-axis, demonstrating that the appearance in pelagic samples was associated with low fish complexity. Chironomids moved to the right along the x-axis, suggesting that when found in pelagic areas they were in lakes of higher salinities.

Several taxa of littoral macroinvertebrates were found in both littoral and pelagic habitats. Based on their different positions (pelagic vs. littoral) in the CCA (Figure 5 insert), chironomids were more frequently encountered in pelagic habitats at higher salinities (shift to more positive values along CCA axis 1), while zygopterans, corixids and amphipods colonized pelagic areas in lakes that had *low complexity* of fish assemblage (shift to more negative values along CCA axis 2).

### Differences among taxonomic groups

For fishes, zooplankton and littoral macroinvertebrates, salinity was the most influential parameter for both richness and species composition. Yet, the thresholds at which communities changed varied (Additional file 1: Appendix 1). All but one fish species (nine-spine stickleback) disappeared from lakes at salinities of more than 2 g L^-1^ (subsaline). For zooplankton, eight of the 20 taxa occurred exclusively in freshwater or subsaline conditions, representing approximately half of the rotifer and cladoceran taxa. In contrast, copepods were much less impacted by salinity as all copepod taxa were encountered in freshwater, subsaline and hyposaline lakes, and two of the four taxa were also found in mesosaline conditions. Other zooplankton taxa either inhabited sub- and hyposaline lakes, or were found only in meso- or hypersaline lakes. For littoral macroinvertebrates, only three of 18 taxa were limited to subsaline conditions (Turbellaria, Hirudinaea and Hydracrina), while the majority of taxa was encountered in both subsaline and hyposaline waters.

## Discussion

Our analyses showed that diversity and food-web composition in prairie lakes was predominantly correlated with salinity as the strongest predictor for richness and taxonomic composition of fishes, zooplankton and littoral macroinvertebrates. Despite this generality, the strength of salinity effects differed among groups. Fishes disappeared quickly with increasing salinity while littoral macroinvertebrates were ubiquitous among lakes. Although zooplankton taxa were also encountered over the whole salinity range, this group showed a clear transition in taxonomic composition along the salinity gradient. Complexity of fish assemblage was also an important determinant of food-web structure. Specifically, the presence or absence of a diverse fish community (*high complexity*) was associated with large changes in zooplankton and littoral macroinvertebrates communities. The directional changes in zooplankton and littoral macroinvertebrate taxa indicated that a more complex fish assemblage was associated with a higher degree of predation pressure on invertebrates. In freshwater lakes with *high complexity* small zooplankton species were dominant, while *low complexity* was not only associated with larger zooplankton taxa but also with littoral macroinvertebrates that colonized the pelagic area. Yet, invertebrate predation that is common in fishless lakes was not observed in these systems [43]. Presumably, as the complexity of fish community decreased, the controlling mechanism of invertebrate assemblage shifted from predation to competition, and ultimately to bottom-up control in hypersaline lakes. The increase in competition in the absence of predation was also supported by the significant negative association of salinity with Chl *a*. As predation is reduced at higher salinities, grazers thrive and can exert a higher pressure on primary producers [44-46].

### Fish

Salinity was most strongly correlated with changes in fish diversity, while nutrients (NO_3_) were of secondary importance (Figure 3). A negative influence of salinity on fish communities has been previously identified [1,47], and is linked to the inability of most freshwater fishes to cope with increasing osmotic stress [3]. With the exception of two lakes (Eduoard and Clair), the richness in freshwater lakes was between four and seven species, and all lakes with salinities below 3 g L^-1^ supported at least one species of fish. Yet, the here observed cut-off point for salinity of < 3 g L^-1^ was fairly low, as most fish species are not only known to withstand higher salinities but were also previously encountered at salinities of up to 6 g L^-1^ in these lakes [1].

Since the surveys conducted in the 1930's and 1960's (summarized in [48] and [1]), regional average annual temperatures have increased by approximately 0.1 °C year ^-1^[49]. Due to the polymictic nature of theses lakes, hypolimnetic cool-water refuges were not available, which may have resulted in increased temperature stress during summer. Moreover, fertilizer use has risen dramatically across the prairies [50], which has resulted in increasing eutrophication. Eutrophic condition are often associated with an elevated risk of winterkill [51] as organic material is being respired at higher rates, especially in shallow lakes that have low dissolved oxygen pools and are exposed to prolonged ice cover during winters [52]. The importance of winterkill in our study lakes is indicated by the fact that the two shallowest freshwater lakes that also had high nutrient concentrations (Edouard and Clair) only supported small fish species known to be more tolerant to oxygen stress [53]. Furthermore, winterkill as a controlling mechanism for fish assemblages in prairie lakes has been reported for several other areas ([54]; [53]. In contrast, stocking programs probably did not have an impact on the fish communities as walleye was only stocked into lakes that were already inhabited by other piscivores (largely yellow perch and pike) to improve recreational fisheries rather than establishing fish populations.

Despite the presence of piscivores (i.e., walleye, pike, and yellow perch) there was nevertheless a predation regime that indicated intense planktivory and benthivory (dominance of small invertebrate taxa). Therefore, the top-down pressure of piscivores on planktivorous and benthivorous fishes was not sufficient to release large zooplankton or conspicuous macroinvertebrates from predation [55]. Furthermore, all piscivorous fish species that inhabited these lakes are planktivorous at least at one point during their life cycles [56,57], and many also rely heavily on benthic prey [24,58], further adding to the predation pressure on large invertebrate taxa.

Lakes of *low complexity* (inhabited by three-spine stickleback or fathead minnows) were characterized by a reduced predation pressure on zooplankton and littoral macroinvertebrates, whereby *low complexity* was encountered in freshwater as well as in hyposaline conditions. Brook stickleback is the most halo-tolerant among fishes and is commonly found in brackish and saline lakes [59,60]. Sticklebacks can be efficient planktivores, often leading to the dominance of small zooplankton species in saline lakes [59]. In contrast, we found that lakes with sticklebacks were usually inhabited by large zooplankton species, indicating that planktivory was either limited to littoral habitats or that stickleback biomass was too low to cause a shift in zooplankton size distribution. In freshwater lakes that had a *low complexity* of fish assemblage, predation pressure on invertebrates was probably even further reduced as these lakes were more likely to suffer winterkills (shallow, high nutrients), reducing survival and reproduction of residing fish populations. Under such circumstances, invertebrate predation often becomes important, and we did observe several large invertebrate predators (e.g., corixids, notonectids) invading the pelagic area. Yet, based on stable isotope analysis, these taxa were not part of the pelagic food web as they continued to rely on littoral diet [13].

### Zooplankton

Zooplankton showed the expected strong changes in species composition along gradients of salinity, which were very similar to previously described patterns in prairie lakes [13]. Additionally, fish assemblage had a strong effect on zooplankton, especially at lower salinities. Small-bodied zooplankton (*D. galeata mendotae, D. rosea, Ceriodaphnia, B. longirostris*) only occupied freshwater lakes that also hosted piscivorous and planktivorous fish (*high complexity*), highlighting small body size as a successful evolutionary adaptation to intense predation pressure of visual predators, such as fish [55,61].

At salinities above 2 g L^-1^, zooplankton occurred in greater abundances and communities consisting of large-bodied cladocerans and copepods in subsaline (*D. pulex* and *L. sicilis*) and mesosaline lakes *(D. similis* and *H. nevadensis*). These species were probably successful in their respective environments because potential invertebrate predators are commonly gape-size limited [62], and a large body size (e.g., *Daphnia* sp.) is a successful defense to escape predation [63]. In addition, larger zooplankton species can be superior competitors over smaller species for limiting food resources [64], [65]). In contrast, a larger body size was an ineffective strategy in most freshwater lakes [66]; [67]) since planktivorous fish are usually orders of magnitude larger than their prey, making it impossible for zooplankton to grow to an invulnerable size [63]. Additionally, in lakes that were fishless or had *low complexity*, zooplankton often exhibited strong pigmentation (Cooper, *pers. observation*), an important protection mechanism against UV radiation, which may be the largest threat in the absence of visual predators [68]. The possibility that salinity was directly restricting the occurrence of large species, such as *D. similis* and *H. nevadensis*, at lower salinities is unlikely because these species can physiologically tolerate freshwater conditions [69,70]. *A. franciscana* was the main zooplankton in hypersaline lakes, which represent a safe refuge from predation, given the lack of physiological adaptation of predators to high osmotic stress. Once both predation and competition become insignificant as controlling factors, population dynamics are likely bottom-up controlled.

Nutrient and Chl *a* concentrations also influenced zooplankton composition independently of salinity and predation (Table 2). Interestingly, greater species richness and population abundances were observed in lakes of lower nutrient levels but higher Chl *a* concentrations, whereby Chl *a* and nutrients were generally uncorrelated among lakes (except the positive association of TKN and Chl *a*, see above). This finding contrasts with prior research suggesting maximum species richness at intermediate productivity [71]. Furthermore, the previous survey by [13] identified a negative association between zooplankton richness and Chl *a*. This discrepancy is likely based on the different ranges in algal biomass among studies and the contrasting effects of high and low algal biomass on species richness ([72]; [73,74]). Across 70 prairie lakes, [13] encountered maximum Chl *a* concentrations that were one order of magnitude higher (negative association with richness) than in this 20-lake study (positive association with richness). The overall negative effects of nutrient levels may be more related to their partial correlation with salinity, rather than bottom-up effects as correlations between Chl *a* and nutrients were weak.

### Littoral macroinvertebrates

Similar to other food-web components, salinity and fish assemblage had significant effects on the taxonomic composition of littoral macroinvertebrates, although the effects were much less pronounced than those observed for zooplankton. Littoral macroinvertebrates were fairly ubiquitous across lakes, with the exceptions of halotolerant taxa, such as chironomids and corixids that became more prominent in lakes between 10 g L^-1^ and 25 g L^-1^, and halophilic *Ephydra* were the only taxon occurring in hypersaline lakes (Figure 5). At lower salinities, concealed taxa occurring within sandy sediments (gastropods, Culicidae, Turbellaria, Hydrophilidae, and Trichoptera) or taxa that use macrophytes as refuge (crayfish, dytiscid, or Ephemeroptera) were found more frequently. As salinity increased and fish were no longer present, littoral corixids, Zygoptera, chironomids, *G. lacustris*, and *H. azteca* were the most common taxa. Additionally, many of these littoral invertebrates were often also found in the pelagic zone (Figure 5 insert). Overall, the observed shifts in taxonomic composition with increasing salinity were likely an indirect effect of reduced predation, rather than a direct response to increased osmotic stress as many of the observed taxa are known to occur over large ranges in salinity [1]. One exception was halophilic ephydrids as the only littoral inhabitant of hypersaline waters. Similar to *A. franciscana*, ephidrids probably benefited from the absence of predation and competition and are likely bottom-up controlled. Beyond salinity, productivity was also identified as a significant variable in determining littoral macroinvertebrate communities. The mechanism was likely similar to that regulating zooplankton (see above), as nutrients and Chl *a* respectively showed the same negative and positive associations for littoral and pelagic diversity.

### Implications for food-web structure

**Figure 6:**
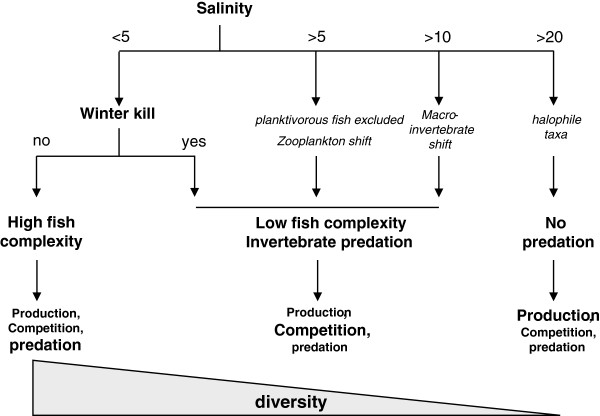
**Conceptual model of the hierarchical relationships among environmental parameters regulating community composition among lakes of the northern Great Plains. **Salinity acts as master variable exerting direct (osmotic stress) and indirect effects (shift in predation regimes). In deep freshwater lakes, predation was likely the dominating factor for zooplankton, with production and competition having minor effects. Mesosaline lakes had low fish predation, leading to increased competition among species. In hyper-saline lakes, there was no predation and the surviving species had a monopoly over the resources; therefore, primary production was the limiting factor. Diversity deceased from freshwater to hypersaline lakes.

Food-web composition in prairie lakes was clearly controlled by a combination of both chemical and biological parameters (Figure 6). As salinity increased from freshwater to hypersaline conditions, taxa were excluded based on their differential sensitivity to osmotic stress. In combination with low water depth, high nutrient levels were likely responsible for an increased probability of winterkill. While we cannot provide any direct evidence for the occurrence of winterkill in individual lakes, previous research showed a clear association between high nutrients in shallow lakes and the potential of winterkill [52,54]; [53]. A second line of evidence for the importance of winterkill in these lakes is that the here identified salinity threshold for exclusion of most fishes was < 3 g L^-1^, which is significantly lower than previously described values of 5–8 g L^-1^ (summarized in [1]). Ultimately, an ongoing study of winter conditions in these lakes will evaluate their susceptibility to winterkill and identify those environmental parameters that are most critical.

In conclusion, piscivorous fishes were most strongly impacted (and eliminated) by adverse environmental conditions, being absent from 13 of 20 lakes. With a reduced complexity of the fish assemblage (and likely significantly lower biomass), predation became less important in structuring pelagic and littoral communities. Instead, competitive interactions among invertebrates in mesosaline lakes and bottom-up effects in hypersaline lakes should have gained in importance as controlling mechanisms (Figure 6).

According to global circulation models, future climate change will result in increased aridity across the Canadian prairies [75]. The ensuing warmer and drier climate will not only reduce freshwater inputs into the lakes but will also increase evaporation. Hence, it is expected that salinity, nutrient levels and water temperatures will rise while water levels will drop, increasing osmotic stress, temperature stress and the probability of winterkill (yet, if winters become milder, the treat of winterkill may actually be reduced due to shorter durations of ice cover). Based on analyses presented herein, lakes that are currently inhabited by piscivorous fishes should undergo the most dramatic changes. In contrast, currently hypo- to hypersaline lakes should be less impacted as ambient taxa are well adapted to endure increases in dissolved substances and fishes are already absent. In respect to taxonomic groups, fishes are most sensitive, while impacts on littoral macroinvertebrates should be minimal due to their ubiquitous nature. For zooplankton, changes in relative importance of individual taxa are likely to occur as this group is most dynamic in its ability to respond to altered environmental conditions. Furthermore, the high diversity and plasticity of zooplankton taxa distinguishes this group to be most suited to continuously assess climate-related changes in prairie lakes.

## Competing interests

The authors declare that they have no competing interests.

## Authors’ contributions

RNC and BW developed the study design and conducted the field operations. RNC conducted all taxonomic analysis, performed statistical analyses, and prepared the initial draft of the manuscript as part of a MS thesis. BW prepared the final draft of the manuscript. All authors read and approved the final manuscript.

## Supplementary Material

Additional file 1 **Appendix 1. **Presence (1) and absence (0) of fishes, zooplankton and littoral macroinvertebrate taxa in the 20 studies lakes during 2007 and 2008. Lakes are ordered by increasing salinity (TDS, g L^-1^), indicated in the last row of the table. Littoral macroinvertebrates that were encountered in pelagic samples are identified as "(litt.)". Click here for file
